# A cost-of-illness analysis of β-Thalassaemia major in children in Sri Lanka – experience from a tertiary level teaching hospital

**DOI:** 10.1186/s12887-020-02160-3

**Published:** 2020-05-27

**Authors:** Hamish Reed-Embleton, Savinda Arambepola, Simon Dixon, Behrouz Nezafat Maldonado, Anuja Premawardhena, Mahinda Arambepola, Jahangir A. M. Khan, Stephen Allen

**Affiliations:** 1 The University of Sheffield, Sheffield, UK; 2 Hemas Hospital, Wattala, Colombo Sri Lanka; 3 Liverpool School of Tropical Medicine, Liverpool, UK; 4 Hemal’s Thalassemia Care Unit, North Colombo Teaching Hospital, Ragama, Sri Lanka; 5 Faculty of Medicine, University of Kelaniya, Ragama, Sri Lanka; 6 National Hospital Kandy, Kandy, Sri Lanka

**Keywords:** Children, Cost-of-illness, Sri Lanka, Thalassaemia

## Abstract

**Background:**

Sri Lanka has a high prevalence of β-thalassaemia major. Clinical management is complex and long-term and includes regular blood transfusion and iron chelation therapy. The economic burden of β-thalassaemia for the Sri Lankan healthcare system and households is currently unknown.

**Methods:**

A prevalence-based, cost-of-illness study was conducted on the Thalassaemia Unit, Department of Paediatrics, Kandy Teaching Hospital, Sri Lanka. Data were collected from clinical records, consultations with the head of the blood bank and a consultant paediatrician directly involved with the care of patients, alongside structured interviews with families to gather data on the personal costs incurred such as those for travel.

**Results:**

Thirty-four children aged 2–17 years with transfusion dependent thalassaemia major and their parent/guardian were included in the study. The total average cost per patient year to the hospital was $US 2601 of which $US 2092 were direct costs and $US 509 were overhead costs. Mean household expenditure was $US 206 per year with food and transport per transfusion ($US 7.57 and $US 4.26 respectively) being the highest cost items. Nine (26.5%) families experienced catastrophic levels of healthcare expenditure (> 10% of income) in the care of their affected child. The poorest households were the most likely to experience such levels of expenditure.

**Conclusions:**

β-thalassaemia major poses a significant economic burden on health services and the families of affected children in Sri Lanka. Greater support is needed for the high proportion of families that suffer catastrophic out-of-pocket costs.

## Background

β-thalassaemia major is Sri Lanka’s most common serious single gene disorder with an estimated prevalence of 2.2% [1, 2]. Medical advances in recent decades have transformed this inherited haemoglobinopathy from a severe, life-limiting disease to a treatable chronic condition. With high quality of care, patients can expect a near-normal life as fully integrated, industrious members of society [2].

The lifelong treatment regime for β-thalassaemia major comprises regular (usually monthly) blood transfusion and iron chelation therapy (ICT) [3]*.* However, despite ICT, transfusional iron overload causes many complications affecting organ systems such as the liver, endocrine organs and heart. Cardiac complications, including pericarditis and dilated cardiomyopathy, still represent 71% of the cause of death in thalassaemic patients [2]. Regular clinic appointments are used to screen for complications including the use specialist equipment such as ultrasound, slit-lamps, audiograms and blood glucose monitoring devices. Blood tests also include serological testing for HIV and hepatitis viruses. The screening and clinical management of these complications requires a specialised multidisciplinary team approach. During hospital care, associated health-care costs include non-medical personnel, staff transport, supplies and requisites, maintenance, electricity, water, food, contractual services and other recurrent expenditure.

Cost-of-illness (COI) studies aim to measure the total societal costs of a disease. Total societal costs extend beyond those related to health care to include household expenditures, and in some studies, lost productivity associated with employment. An important concept related to COI studies is the “cost burden” of a disease, which refers to household cost expressed as a percentage of household income. A common approach to measuring economic hardship associated with health payments is to define a ‘catastrophic’ spending level of > 10% of household income. This degree of cost burden is considered to directly impact consumption of basic needs such as food and education or trigger the sales of assets leading to higher levels of debt or poverty [4–8].

We identified 14 COI studies of thalassaemia, published between 1975 and 2017, in Canada, India, Iran, Israel, Italy, Myanmar, Taiwan, Thailand and the UK [1, 7–19]. Reported medical costs to health services ranged from $US 873 to almost $40,000 per patient year [9, 13]. In Sri Lanka, the national blood transfusion service is provided by the Ministry of Health and comprises 98 hospital-based blood banks and there are two standalone thalassaemia centres. De Silva et al. estimated in 2000 that the cost of preparing blood, ICT, essential investigations and hospital visits was LKR 175,000 (equivalent to $US 2465) [3]. This estimate is now outdated in view of substantial changes to patient care.

The costs of thalassaemia do not just fall on the health service but also on the affected individual and their household as treatment decisions and coping mechanisms usually occur at the household level [20]. Seven of the economic analyses assessed costs to the household [1, 7, 9, 11, 16, 18, 19] but none assessed the cost burden. No assessment of the costs to families was undertaken in the study in Sri Lanka [3]. We undertook a prevalence based, cost-of-illness study to provide an updated estimate of the economic burden to both the health service and families of transfusion dependent β-thalassaemia in children in Sri Lanka. Health service costs include both direct hospital costs, which are directly related to patient care such as staff costs, and indirect hospital costs, such as overheads. Household costs include items such as travel and food costs when attending treatment centers.

## Methods

### Study location

This study was undertaken in Kandy Teaching Hospital (KTH) in the Central province of Sri Lanka. KTH, the second largest medical institution in the country, has two main paediatric wards with a capacity of 100 beds and a single integrated 8 bedded blood transfusion unit. As well as patients living in the hospital catchment area, many patients from adjoining districts attend for specialised medical care such as thalassaemia management. In 2017 there was over 22,000 admissions to KTH including almost 8000 paediatric cases [21].

### Patients

The inclusion criteria were children (< 18 years) with a diagnosis of β-thalassaemia major who had attended KTH for at least 1 year**.** All children who attended for blood transfusion during the period of 12th June – 11th of July 2017 were invited to take part in the study. In order to estimate the mean cost of the patients and assuming a normal distribution, we aimed to recruit at least 30 cases as directed by the Central Limit Theorem [22].

### Collection of demographic and clinical data

At the time of blood transfusion, demographic data and the number of transfusions and units of blood received, investigations and drug treatment over the preceding 12 months was extracted from case records.

### Estimating health service costs

Costs were estimated for both in-patient care and attending out-patient clinics. Staff costs per inpatient day were calculated using estimates of workload intensity [23]. Staff costs were allocated to individual patients based on the complexity of their care needs categorised according to a four point scale, with each category representing a measure of workload intensity (Table 1). Patients were allocated to the scale by the doctors working on the relevant wards.

**Table 1:** Workload unit scoring system

Definition	Patient score	Relative workload intensity
Patients who require less than average care E.g. Regular transfusion visits	1	1
Sub-acute patients who require the standard level of care	2	2.5
Acute patients who require more than average care	3	3.5
Intensive care patients who require a high level of care	4	7

A breakdown of monthly overhead spending for the hospital was provided by the accounting staff. Direct hospital costs were then inflated to take into account the indirect costs of care using the mark-up method [24]. With this method, the ratio of indirect to direct costs is calculated based on available budget information, then used to adjust the direct costs associated with the patient population of interest (and for which indirect cost information is not available) providing an estimate of the total hospital cost (direct + indirect).

The equipment used for monitoring for complications was not exclusively used in the care of thalassaemic patients; therefore, we estimated the cost per test of using such equipment. The price of equipment along with its estimated life expectancy was used to calculate the equipment cost per test. The cost per month of equipment (mE) was calculated using the formula $\mathrm{mE}=\frac{\mathrm{cE}}{\mathrm{L}}$, where cE is the purchase price of equipment and L is the life expectancy in months. The cost per test (C) was calculated using the formula $\mathrm{C}=\frac{\mathrm{mE}+\mathrm{sW}}{\mathrm{n}}$, where sW is the monthly staff wages required to run the clinic and n is the total number of tests performed in 1 month. Table 2 summarises the resource consumption and measurement.

**Table 2:** Summary of resource consumption

Resource	Measure	Source of data	Valuation
**Blood transfusion**			
- Staff	Time spent	Accounting department	Salary
- Transfusion consumables	Number and types of transfusion	Patient records Pharmacy department Blood bank	Price per item
**Drug therapy**			
- ICT	Dose and frequency	Patient records	Price per item
- Concomitant medication		Pharmacy department	
**Clinic and outpatient**			
- Staff	Number of type	Patient records	Salary
- Equipment		Clinician interviews	Cost per test
**Overheads**	Number of items of shared services	Accounting department	Price per item
**Household costs**			
- Transport and food	Expenditure per visit	Structured interviews	Self-reported

### Estimating household costs

Structured interviews were conducted with children and their parents/guardians in the local language by a trained research assistant (see interview guide in Additional file 1). We expressed household health expenditure per month as a percentage of total monthly household income [25–28].

### Statistical analysis

Descriptive statistics were used to summarise the participants and cost items. Mean (SD) was used for normally distributed data and median (IQR) for non-normal data. A Pearson correlation test was used for analysis and *P*-value < 0.05 for statistical significance.

## Results

All participants who were invited to join the study agreed to take part and a total of 34 children attending for blood transfusion were enrolled. Median age was 10.0 years (range 2.3–17.0 years) and 22 (64.7%) were female. Median (range) age at first transfusion was 4 [1–11, 29, 30] months (age not available for one child).

### Direct hospital costs

#### Blood transfusion and ICT

Median (range) number of transfusion sessions per year was 12.0 (11.0–14.0) and the median (IQR) number units of units transfused per year was 21.0 (18.5–23.0). The cost of preparing 1 unit of blood was $US 44 (personal communication; Head of the Blood Bank, KTH) resulting in an average cost of $US 893 per patient year for blood transfusions.

26 (76.5%) patients received oral deferasirox, 1 (2.9%) IV deferoxamine and 7 (20.1%) combined ICT including transition from oral to IV treatment. During the 12 months studied, 27 patients remained on a stable dose of ICT whilst 7 patients changed either their dose or ICT agent at least once. The cost of oral deferasirox 100 mg and 400 mg was $US 0.61 and $US 1.34 respectively and IV deferoxamine 500 mg cost $US 3.04. On average, ICT cost was mean (SD) $US 967.3 (651.7) per patient year. The average cost of concomitant medication was mean (SD) $US 5.10 (7.2) per patient year. ICT accounted for 99.5% of the total drug costs.

#### Staff

The paediatric team was composed of 1 Consultant, 1 Senior Registrar, 3 Registrars, 4 Senior House Officers, 4 House Officers, 1 Sister and 17 Nurses. The combined monthly salary of these staff was $US 14,126. The total workload units were 107 which gives a staff cost of $US 4.34 per workload unit. Patients remained on the ward for a mean (SD) of 2.1 (0.54) days per admission and this amounts to an average staff cost of $US 114 per patient year.

#### Investigations and clinic visits

The number of investigations, costs per unit and cost per patient year of each investigation were combined with clinic attendance costs; totalling $US 3832 equivalent to $US 113 per patient year***.***

**Fig. 1:**
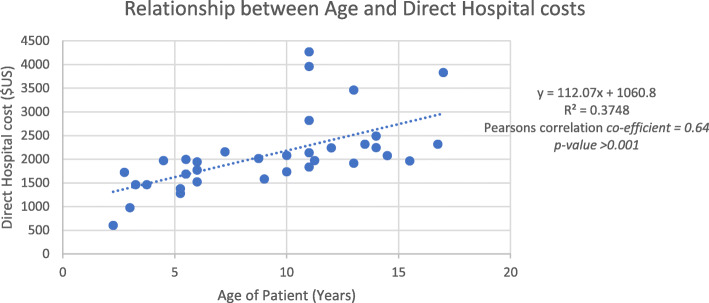
Relationship between age and direct hospital costs. y = 112.07x + 1060.8 R^2^ = 0.3748. Pearsons correlation co-efficient = 0.64

Age positively correlated with treatment cost (*p*-value< 0.001) (Fig. 1) reflecting the increased transfusion and ICT requirements in older children with an increase of $US 112 for every 1 year increase in age.

### Indirect hospital costs

#### Overhead and building

The total indirect costs were $US 389,510 and the total direct costs borne by the hospital were $US 1,600,910 giving a mark-up percentage of 24.3%. This equates to an additional $US 509 per patient year in overhead costs.

### Household costs

In 31 (91.2%) children, the mother provided information, the grandmother in 2 (5.8%) and the father in 1 (2.9%). The highest educational level for household head was primary for 6 (17.6%), secondary for 24 (70.6%) and graduate for 3 (8.8%; not reported for 1 child). Four (11.8%) of the respondents were self-employed, 1 (2.9%) was employed and 29 (85.3%) reported housework as their occupation.

Total household costs were $US 206 per year with food and transport ($US 99 and $US 51 respectively) being the highest cost items. Two patients reported hospitalisation in the past 12 months; 1 for 15 days and 1 for 4 days with a household cost of $US 164 and $US 3 respectively which was included in the household costs. The other cost items measured can be found in the supplementary file. Eight children were too young to attend school and the remaining 26 reported median (range) of 37.5 (24–84) days of absence from school per year.

#### Household cost burden

**Fig. 2:**
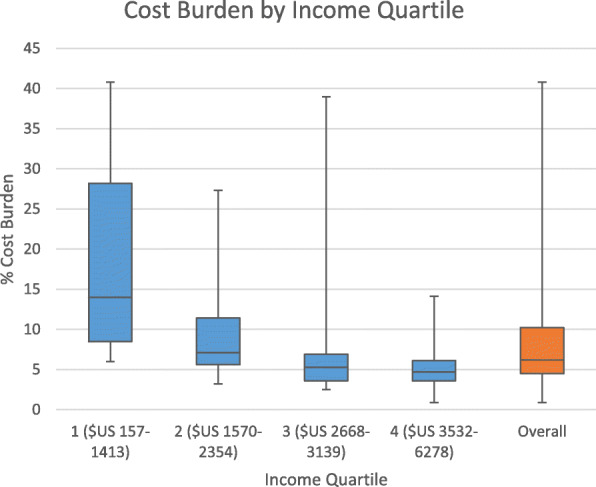
Relationship between income quartile and cost burden. Boxes show Median and IQR, whiskers show range

Mean (SD) household annual income was $US 2548 (1340). One household reported no income being dependent on bank loans and was excluded from the cost burden calculations as their income denominator was 0. Figure 2 shows that cost burden varied between income quartiles. Only 1 household had a low-cost burden and 9/33 (27.3%) experienced a catastrophic cost burden despite the free medical care available in Sri Lanka. Households in the lowest income quartile experienced a median cost burden of over 10% which is often regarded as ‘catastrophic’ [4–6]. Of households in the lowest income quartile, cost burdens were either high (*N* = 2) or catastrophic (*N* = 5).

In total 5 families took out a loan to help cover the costs generated by their child’s thalassaemia. Of these 4 out of 5 were in the lowest income quartile (one was in the second highest).

### Total costs

**Fig. 3:**
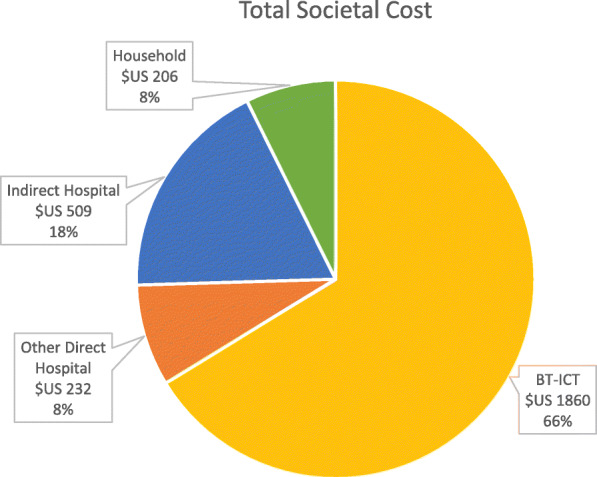
Total societal cost. Values are reported in $US and expressed as a percentage of the total societal cost

The total annual direct hospital cost was $US 2092 per patient year. This figure was inflated accordingly by the mark up of 24%, which amounts to $US 2601 per patient year. Household costs were $US 206. This amounts to a total societal burden of $US 2807 per patient year. Figure 3 reports the breakdown of total societal cost expressed as a percentage. As shown BT-ICT makes up the 66% of the total cost.

## Discussion

This cost-of-illness study of transfusion dependent β-thalassaemia major in children in Sri Lanka includes a comprehensive assessment of direct and indirect costs to the healthcare system and costs to household budgets. We estimated that the healthcare costs of managing thalassaemia are approximately $US 2601 per patient year. In addition, despite free healthcare in Sri Lanka, households frequently spend over 10% of their annual income on blood transfusion sessions, follow up tests, special foods and hospitalisation; with those most likely to spend this ‘catastrophic’ level being from the lowest income quartile.

The only previous economic analysis in Sri Lanka, published in 2000, estimated an annual cost of treating thalassaemia of LKR 175,000 ($US 2465) per patient year [3]. When inflated by the consumer price index provided by the World Bank [31] this value is $US 10,649 at present day value. One potential cause for this lower cost is in the change in the drug of choice for ICT from a sub-cutaneous infusion of deferoxamine to oral deferasirox. Deferasirox is considerably cheaper than IV deferoxamine at current pharmacy prices (500 mg = $US 1.96 vs 3.06; costs provided by the pharmacy department) and does not require an infusion pump for administration. Other advances in medical care likely also contribute to this lower estimate. The International Diabetes Federation (IDF) estimated the cost of managing diabetes in Sri Lanka at $US 185 per patient year in 2017 [32]. This means we estimate the annual direct hospital cost of thalassaemia in children ($US 2092) to be over 10 times that of an adult diabetic patient.

Factors accounting for the variation in costs in previous studies [1, 7–19] included age of the patients, treatment regimen and the number of complications. Differences in study design included the use of hypothetical patients and which costs were included such as productivity loss and those related to complications. Costs varied considerably between studies conducted in the same country. For example In Iran, the cost per patient year ranged from $1730 to $8321 [18, 19]. This highlights the disparities in methods of cost assessment including the use of assumptions related to treatment patterns, patient sample, overhead allocation method, local unit costs and data collection methods; all of these factors limit the scope for inter study comparison. The use of a standard set of reporting guidelines as recommended for cost-effectiveness studies [33] would ensure that studies capture similar costs and enable better inter-study comparison.

The true economic impact of a disease must consider factors beyond health related expenditures, such as family coping strategies and impact on future livelihood [20]. In severe poverty, where a household struggles to achieve minimum food or fuel levels, even a small change (e.g. the loss of 1 day’s wage) may have substantial implications for the wellbeing of the whole household requiring drastic coping strategies [4]. Family strategies often aim to maintain short-term economic sustainability for the household [34] but the selling of assets or borrowing of money to help with treatment clearly generate future challenges and costs. The relatively low levels of household income in our study resulted in about 1 in 4 households experiencing catastrophic costs (> 10% of total income). Of the 5 families who took out a loan to help cover the costs of thalassaemia, 4 were from family or neighbours exemplifying the bonding / bridging forms of social capital, and the greater need for the poorest families to utilise family networks and assets compared to families with more resources [35]. To further understand the complex dynamics of household expenditure on healthcare and the impact of lost schooling for individuals, longitudinal in-depth quantitative and qualitative research that assesses expenditure and future income implications is needed. A greater understanding of coping methods and how assets are mobilised is necessary to assess the impact on household livelihoods. We found out-of-pocket costs were not associated with household income (Pearson coefficient 0.21, *p*-value 0.20). Since the out-of-pocket payments contained mainly transport and food costs, the observed correlation was not unexpected. Our results indicate that there are discrepancies between household cost burden across income quartiles, despite the free medical care available in Sri Lanka. However, we were unable to adequately investigate spending patterns and no data were collected on household’s ability to pay for basic needs after the cost of healthcare had been taken out. Studies that explore how households prioritise expenditure, how they perceive basic needs, and the factors which underpin inequality and financial protection would help generate a more complete picture.

### Strengths and limitations

The study estimated the costs of managing β-thalassaemia major both for health services and households. We recruited children of diverse ages, socioeconomic backgrounds and healthcare requirements to provide a broad view of costs to households. However, the data relate only to children in one specific healthcare setting; conducting the study in more than one hospital site and recruiting a greater sample size, would improve validity and generalisability especially as distance from treatment facilities and, therefore, transport costs may differ amongst regions. This additional information would be important when considering policy implications at the national level. In 2016, the median income in Kandy was 42000Rs compared with a national median of 44000Rs as reported by the Department of Census and Statistics [36]. Kandy was the 4th richest of 25 districts in this census data with the national median somewhat skewed by Colombo which has a median of 70,000Rs. Therefore, we consider that the economic status of the Kandy population is approximately representative of the national population outside of Colombo.

When calculating the mark-up percentage, sufficient data were not available to determine whether certain cost centres were utilised by the patients in our study. The high cost of medication for ICT is unlikely to have a linear correlation with overhead costs as assumed when using the marginal mark-up methodology. This means the mark-up percentage may be an over-estimate of the actual value. On the other hand, the cost of administrative, domestic and pharmacy staff were not included in the overall staff cost. A study of administrative costs in 8 nations reported administrative costs make up between 12 and 25% of total hospital costs [37].

In this study, costs were only estimated over a one-year period. Longer periods of observation are needed to get a more accurate view of costs and to quantify the long-term consequences of thalassaemia. This is of particular importance in thalassaemia due to the increasing cost with age as demonstrated in other studies. Using an incidence-based approach (which includes costs throughout a patient’s lifetime) would be more useful in policy decision making where preventative measures are considered as it provides a more accurate level of saving. Also, incidence-based studies allow an analysis of the disease throughout the life-course allowing researchers to develop improved clinical and therapeutic guidelines for disease management.

Finally, we did not attempt to estimate the cost of the potential impact that loss of education could have on future financial capabilities and acquisition of household human capital [38]. A study comparing adult employment and income in people with β-thalassaemia and those without would be required. This could then be applied to the number of adults with β-thalassaemia in a prevalence-based COI study.

## Conclusion

Managing thalassaemia cost the healthcare system in Sri Lanka an estimated $US 2601 per patient year. Most of this total cost can be attributed to blood transfusion and ICT. Despite free healthcare, many households incurred catastrophic costs. Many families caring for a child with β-thalassaemia require financial support to mitigate adverse financial hardship.

## Supplementary information

**Additional file 1.**

## Data Availability

The datasets analysed during the current study are available on request from the corresponding author.

## Abbreviations

COI: Cost of illness
ICT: Iron chelation therapy
IQR: Interquartile range
IV: Intra venous
KTH: Kandy Teaching Hospital
LKR: Sri Lankan Rupees
SD: Standard deviation
UK: United Kingdom
$ US: United States dollar
