# Supplementary material for: A cost-of-illness analysis of β-Thalassaemia major in children in Sri Lanka – experience from a tertiary level teaching hospital
Source: BMC Pediatr. 2020 May 27;20:257. doi: 10.1186/s12887-020-02160-3 (PMC7251920; doi:10.1186/s12887-020-02160-3)
Supplement: Supplementary file 1 — Additional file 1. [file 12887_2020_2160_MOESM1_ESM.docx]

**Patient interview**

Questionnaire Number:

Patient Registration Number:

Date of Interview: Location of interview:

| **Patient Information** | | |
| --- | --- | --- |
| 1. **Gender**  Male Female | | **Age of Patient (Years/Months):** |
| 2. **Age at diagnosis (Years/Months):** | **Age starting regular transfusions (Years/Months):** | |
| 3. **Patient Height (cm):** | | **Patient Weight (Kg):** |
| 3. **Other diagnoses (If known):** Unknown  HIV Hepatitis B Hepatitis C Diabetes Osteoporosis Hypersplenism  Hypothyroidism Hypoparathyroidism Cardiomyopathy | | |

| **Respondent Information** | |
| --- | --- |
| 4**. Gender** Male Female | **Age (Years/Months):** |
| 5**. Occupation:** | **Relationship to patient:**  Parent Supporter  Other (Specify) |
| 6.**a) What district do you live in?**  **b) Is there another transfusion centre closer to you?**  **c) If YES why do you not use that centre ?** Stigma Better service Other (Specify) | |
| 7. **Why does somebody accompany your child?**  Distance Security Administrative barriers Too ill to travel alone  Required for treatment Young child Other (specify): | |

| **Socioeconomic Information** | | |
| --- | --- | --- |
| 8**. Who is the primary income earner in the household?**  Patient Wife/Mother Husband/father Extended family Son/Daughter  Other (Specify) | | |
| **What is the highest level of education of the…?**  **9. Patient**  Graduate Secondary Primary Not attended/illiterate Other:  **10. Primary income earner**  Graduate Secondary Primary Not attended/illiterate Other  **11. Head of household** Same as above  Graduate Secondary Primary Not attended/illiterate Other  **12. Spouse of the head of household**  Graduate Secondary Primary Not attended/illiterate Other | | |
| 13. **Are you currently formally employed?** | - Yes, formal work - Go to 16  - No, informal work –Go to 16  - On leave  - Retired  - School / University  - Housework  - Combination (Specify)  - Other (Specify) | |
| **14. Is your reason for not working related to your child’s thalassaemia?** | | Yes No |
| **15. If YES: When were you last working (mm/yy)** | |  |
| **16. What is your personal income per month** | |  |
| **17. What is the estimated income of your household per month currently?** | |  |
| **18. What was the estimated income of your house per month before your child was diagnosed with thalassaemia?** | |  |
| If 16 and 17 differ:  **19. Is the change related to your child’s thalassaemia** | | Yes No |

| **Treatment costs** | | |
| --- | --- | --- |
| **Costs related to Transfusion** | | |
| **20. How often does your child undergo transfusion?** | Fortnightly Monthly  Other (Specify) | |
| **21**. **How long does it take you to get there? (One way)**  ­­__________Hours walking __________Hours with transport ­­­­­­__________Other: | | |
| 22. **What is the total turnaround time Door to Door from leaving your home to getting back to your home after transfusion?** | |  |
| **23. a) From your home to the hospital and back to your home again how much does it cost to take transport?**  **b) How many days do you spend in hospital for transfusion** | |  |
| **24. How much do you spend on food on the road when coming for transfusion?** | |  |
| **25. a) Does anyone else ever attend transfusion? Who?**  **b) What extra costs do they have? For food and travel / visit** | |  |
| **26. Do you have any accommodation costs when coming for your child’s transfusion?** | | No Yes  **How much?** |

| **Costs related to follow up tests – ICT clinics, Cardiac echo monitoring, Abdominal USS, Hearing clinic, Visual clinic, Ferritin elsewhere** | |
| --- | --- |
| **27. Do you ever have to visit a health facility in relation to thalassaemia in addition to transfusion?** | Yes _________/year  No |
| **28. Do you have to pay any additional costs for these?** | Yes No |
| **29. How much for…?**  ­­Fees__________ Tests__________ Drugs__________ Other__________ | Total /Visit__________ |
| **30.** **How long does one of these visits take on average, including time on the road and waiting time? (total turnaround time)** |  |

| **Hospitalisation – Not including transfusion visits!** | |
| --- | --- |
| **31. Has your child been hospitalized in the past 12 months related to thalassaemia?** | Yes _________/year  No – Go to 46 |
| **32. How many days were they in hospital?** | __________Days |
| **33. How did you pay during the stay for …?**  ­­Fees__________ Tests__________ Drugs__________  Bed __________ Transport__________ Food__________  Other__________ | Total /Visit__________ |
| **34. Did someone stay with your child while in hospital?** | Yes  No – Go to 44 |
| **35. How many days did they sleep there?** | __________Days |
| **36. Where there any extra costs to stay in the hospital**  Accommodation­__________ Food__________  Transport___________ Other __________ | Yes  Total __________  No |
| **37. Did anyone else visit your child while in hospital**  **a) How many people?**  **b) How many times?**  **c) Costs**  Accommodation__________ Food__________  Transport___________ Other __________ | __________People  __________Person times  __________Total |
| **38. How long were the visits including travelling time?** |  |

| **Other costs** | | | | |
| --- | --- | --- | --- | --- |
| **Food supplements** | | | | |
| **39. Do you buy any supplements for your child’s diet because of thalassaemia?**  b) Fruits Drinks Vitamins/Herbs Meat  Other (specify) | | | Yes  No | |
| **40. How much did you spend on these items in the last month?** | | | __________Total/Month | |
| **Other illnesses** | | | | |
| **41. Are there any additional costs for chronic illnesses related to thalassaemia that you have not already mentioned?**  **­­**Fees__________ Tests__________ Drugs__________  Bed __________ Transport__________ Food__________  Other__________ | | | Total__________/Month | |
| **Coping costs** | | | | |
| **42. Do you borrow any money to cover the costs of thalassaemia?** | | | | Yes  No Go to 55 |
| **If YES**  **43. a) Who did you borrow from**  **b) How much?** | Family Neighbour  Bank Other (specify)  __________Total/year | | | |
| **44. What is the interest rate on the loan?** | | __________%  I am not expected to pay back the money | | |
| **45.a) Have you sold any of your property to finance the cost of thalassaemia?** | | | Yes  No – Go to 56 | |
| **b) What did you sell?**  **c) How much did you earn from the sale of your item(s)**  **d) What was the market value of these item(s)** | | Land Livestock Vehicle Household item Farm produce Other (Specify)  __________  __________ | | |

| **Productivity loss -** | | | | |
| --- | --- | --- | --- | --- |
| **46. a) Has your child stopped going to school due to thalassaemia in the past year? – INCLUDES TRANSFUSION**  **b) How long for?** | | | | Yes No – Go to 49  ___Months___Weeks___Days |
| **47. a) Does someone stay at home specifically to look after them?**  **b) If YES: For how long?**  **c) Did they quit their income earning job to stay at home?** | | | | Yes No - Go to  ___Months___Weeks___Days  Yes No |
| **48. How regularly did you work before your child was born with thalassaemia?** | | | Throughout the year Seasonal/part  Day labour Other | |
| **49. Did you have to change jobs after your child was born?** | | | | Yes No |
| **50. What is your main occupation**? | | Sales/service Agriculture Household Production/construction Combination (specify)  Other (specify) | | |
| **51. How many hours did you work on average per day BEFORE your child was born?** | | | | ______Hours |
| **52. How many hours did you work on average per day AFTER your child was born?** | | | | ______Hours |
| **If 61 / 62 differ:**  **53. Is this change related to thalassaemia?** | | | | Yes  No |
| **54. a) Do you have other children of school age?**  **b) Do these children attend school regularly?** | | | | Yes No – Go to 65  Yes – Go to No |
| **c) If NO: Why not?** | Housework No money for school Also sick  Works for income to finance costs of thalassaemia Other (specify) | | | |
| **55. a) Has thalassaemia affected your social/private life in any way?**  **b) Has this resulted in financial burden** | | | No – Go to  Divorce/Separation Loss of job Dropped out of school Disruption of sexual life  Other (specify)  Yes No | |
